# Complete Mitochondrial Genome of *Gyrodactylus nigeri* (Platyhelminthes: Monogenea)

**DOI:** 10.1002/ece3.73049

**Published:** 2026-02-10

**Authors:** Yuhan Yang, Yang Liu, Wenhan Yue, Yuxuan Chen, Man Kang, Yulin He, Tao Chen

**Affiliations:** ^1^ Guangxi Key Laboratory of Diabetic Systems Medicine Guilin Medical University Guilin People's Republic of China; ^2^ School of Basic Medical Sciences Guilin Medical University Guilin People's Republic of China; ^3^ Key Laboratory of Pathogenic Biology (Guilin Medical University) Education Department of Guangxi Zhuang Autonomous Region Guilin People's Republic of China; ^4^ School of Basic Medical Sciences Dali University Dali People's Republic of China

## Abstract

*Gyrodactylus nigeri* Zhou & Chen, 2024 was merely distributed in Yunnan Province, Southwest China; meanwhile, its mitochondrial genome remains unclear. This study aims to sequence the mitogenome of *G. nigeri* and clarify its phylogenetic relationship within the Gyrodactylidea. The mitogenome of *G. nigeri* was sequenced using the next‐generation sequencing (NGS) method, annotated, and analyzed using bioinformatic tools. The mitochondrial genome of *G. nigeri* is 14,903 bp in length, containing 12 protein‐coding genes (PCGs), 22 transfer RNA genes (tRNAs), two ribosomal RNA genes (rRNAs), and two major non‐coding regions (NCR: NC1 and NC2). The overall A + T content of the mitogenome is 76.6%, a higher content compared with all reported mitochondrial genomes of monogeneans. The mitogenome of *G. nigeri* presents a clear bias in nucleotide composition with a negative AT‐skew and a positive GC‐skew. All tRNAs have the typical cloverleaf secondary structure except for *tRNA* ^*Cys*^, *tRNA* ^*Ser1*^, and *tRNA* ^*Ser2*^, which lack the dihydrouridine (DHU) arm. Furthermore, two different repetitive non‐coding regions of 88 bp repeats occurred in the NCR regions (NC1 and NC2) with a poly‐T stretch, two stem‐loop structures with obvious differences in the first loop, and a G(A)n motif. The gene order is identical to the mitochondrial genomes reported from other *Gyrodactylus* species except *Gyrodactylus* sp. FZ‐2021. Co‐phylogenetic analyses showed phylogenetic divergence patterns of *Gyrodactylus* correspond to those of their fish hosts, and the overall coevolutionary fit between the parasites and hosts was consistently significant. Meanwhile, the results supported the sister relationship between *G. nigeri* and *Gyrodactylus* sp. FY‐2015 from the hosts within the Nemacheilidae cluster together with high nodal support based on 12 PCGs sequences and amino acid sequences. Gyrodactylidae forms an independent and monophyletic clade within Gyrodactylidea.

## Graphical abstract

The fish *Yunnanilus niger* is the host of *Gyrodactylus nigeri*. The mitochondrial genome of *G. nigeri* is 14,903 bp in length. Phylogenetic analyses indicated that this species belonged to the genus Gyrodactylus and Gyrodactylidae forms an independent monophyletic clade within Gyrodactylidea.

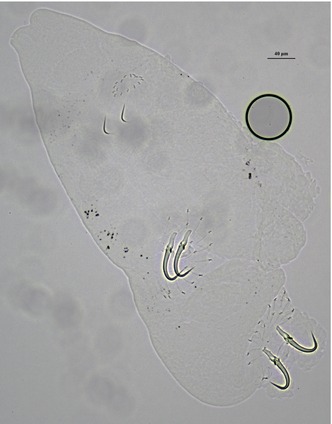

## 1 Introduction

The black loach *Yunnanilus niger* Kottelat & Chu, 1988 is merely distributed in a tributary of the Nanpanjiang River upstream of the Pearl River drainage of Yunnan Province in Southwest China and is distinguished by its deep black body and fins, except for the caudal fin (Kottelat and Chu 1988). Because of its narrow habitat distribution, limited dispersal ability, and geographical isolation, studies on the endemic species *Y. niger* have mainly focused on morphological identification (Kottelat and Chu 1988; Chen 2013), mitochondrial genome (GenBank number MW553077, unpublished), phylogenetic relationships (Du et al. 2023), and parasite description (Zhou and Chen 2024). Recently, the unique *Gyrodactylus* species *Gyrodactylus nigeri* Zhou & Chen, 2024 was recorded on *Y. niger* in Southwest China (Zhou and Chen 2024).

Gyrodactylidea is a hyperdiverse monogenean order with more than 610 described species (Bakke et al. 2007; Kritsky et al. 2020; Ciccheto et al. 2023; Lebedeva et al. 2023; Rahmouni et al. 2023, 2025; Shigoley et al. 2023; Ondračková et al. 2023; Benovics et al. 2024; Jin et al. 2024; Hao et al. 2024; Zhou and Chen 2024; García‐Vásquez et al. 2025; Vancheva and Georgiev 2025). However, merely fourteen mitogenomes of Gyrodactylidea parasites were sequenced, including thirteen species of Gyrodactylidae and one species of Oogyrodactylidae (Huyse et al. 2007, 2008; Ye et al. 2014, 2017; Zou et al. 2016; Zhang et al. 2016; Bachmann et al. 2016; Vanhove et al. 2018; Zeng et al. 2025). These studies found the gene arrangement and tandem repeat units of Gyrodactylidea species, including parasites *Cichlidarus nyanzae* (Paperna, 1973), Janulewicz, Pietkiewicz & Ziętara, 2024, and *Macrogyrodactylus karibae* Douëllou & Chishawa, 1995 in Africa (Vanhove et al. 2018; Janulewicz et al. 2024), *Gyrodactylus* sp. FZ‐2021 (unpublished), *Paragyrodactylus variegatus* You, King, Ye & Cone, 2014 in Asia (Ye et al. 2014), and *Aglaiogyrodactylus forficulatus* Kritsky, Vianna & Boeger, 2007 in South America (Bachmann et al. 2016). In addition, there is a tandem repeat unit in *Gyrodactylus pseudorasborae* Ondračková, Seifertová & Tkachenko, 2023 in Southwest China (Zeng et al. 2025). The A + T content of whole mitogenomes and their elements within the order Gyrodactylidea ranged from 62.5% ( *Gyrodactylus salaris* Malmberg, 1957) (Huyse et al. 2007) to 80.1% ( *C. nyanzae* ) (Vanhove et al. 2018; Janulewicz et al. 2024), while the mitogenome of *Paratetraonchoides inermis* Bychowsky, Gussev & Nagibina, 1965 was the highest A + T content (82.6%) among the monogenean (Zhang et al. 2017). As *Y. niger* is listed as Vulnerable (VU) in the International Union for Conservation of Nature (IUCN) red list status (https://www.iucnredlist.org), it is considered to be facing a high risk of extinction in the wild. Although the new species *G. nigeri was* identified recently in Southwest China (Zhou and Chen 2024), its mitogenome is unreported, so it is interesting to test gene arrangement and tandem repeat units of the mitogenome of *G. nigeri* in the Oriental realm and compare its phylogenetic relationship within Gyrodactylidae, which is important for host protection. Therefore, we aimed to sequence and obtain the entire mitogenome of *G. nigeri* using the next‐generation sequencing (NGS) method. The findings will enrich the molecular database within the Gyrodactylidea and be used for host protection. Specifically, we analyzed the co‐phylogenetic relationships among complete mitogenomes in Gyrodactylidea and fish host to illustrate the evolutionary relationship of Gyrodactylidae and explore whether phylogenetic divergence patterns of *Gyrodactylus* correspond to those of their fish hosts.

## 2 Materials and Methods

### 2.1 Ethical Approval

The study was approved by the Animal Care and Use Committee of Guilin Medical University (Accession number: GLMC202303011). In addition, the method of euthanasia on fish and parasites was performed in accordance with the American Veterinary Medical Association (AVMA) guidelines for the euthanasia of animals (2020). The fish were anesthetized with 0.02% MS‐222, and their body surfaces were examined for gyrodactylids using a stereoscopic microscope. The black loach *Y. niger* is listed as Vulnerable (VU) in the International Union for Conservation of Nature (IUCN) red list status (https://www.iucnredlist.org) and not endangered or protected in China (Yue and Chen 1998). Fish sampling was permitted by the local level authority for scientific research.

### 2.2 Sample Collection and DNA Extraction

**FIGURE 1:**
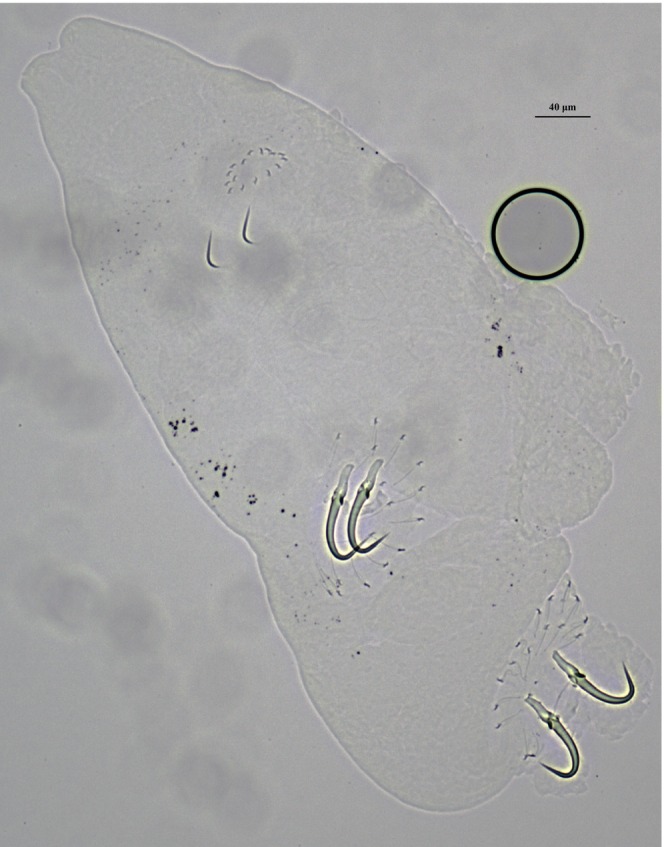
Whole mount specimen of *Gyrodactylus nigeri*, scale bar: 40 μm.

In this study, the gyrodactylid was collected on *Y. niger* by hand netting and a net shrimp cage in the Dalubei from Qujing City of Yunnan Province, Southwest China (25.43° N, 103.75° E; altitude 1931 m) in January 2024 and identified based on their morphological characteristics (Kottelat and Chu 1988). Five specimens of *G. nigeri* were deposited at the Museum of the Institute of Hydrobiology, Chinese Academy of Sciences, Wuhan City, Hubei Province, China (http://www.ihb.ac.cn/) under the voucher numbers YN‐GN202401‐05 based on a previous study (Zhou and Chen 2024). Previous studies, based on the morphometric identification and sequencing of internal transcribed spacer (ITS) rDNA, have shown that *G. nigeri* is the only species found on the host in the studied area (Zhou and Chen 2024) and added its whole morphological image (Figure 1). Total genomic DNA was extracted from the body of one specimen following the operation instructions of the TIANamp Micro DNA Kit (Tiangen Biotech, Beijing, China). The degrees of concentration and purity of DNA were evaluated using a Thermo Scientific NanoDrop 2000. Finally, the specimens of host and *G. nigeri* were stored in 95% ethanol, respectively.

### 2.3 Mitochondrial Genome Sequencing and Assembly

The mitogenome sequencing and assembly were performed by Sangon Biotech (Shanghai, China). Next‐generation sequencing was used to sequence the entire mitogenome of *G. nigeri* from a single individual. First, an Illumina library was built using iTru adaptors and primers, as well as the KAPA Hyper Plus kit, according to the manufacturer's protocol (Glenn et al. 2019). The library was then sequenced using an Illumina HiSeq 4000 PE150. Furthermore, the raw reads were evaluated and cleaned to benefit from FastQC and Trimmomatic, respectively (Bolger et al. 2014; de Sena Brandine and Smith 2019). Finally, the mitogenome was assembled using GetOrganelle v1.7.7.1 (Jin et al. 2020). Furthermore, De Novo Assemble, Map to Reference, and BLAST were used to verify the result of the mitogenome in accordance with Geneious 11.1.2 (Ripma et al. 2014).

### 2.4 Mitochondrial Genome Annotation and Analysis

The MITOS model was used to predict the locations of protein‐coding genes (PCGs) and rRNA genes (RNAs) (Al Arab et al. 2017; Donath et al. 2019). The initiation and termination codons were identified using an open reading frame (ORF) finder and Blastn of NCBI, according to their alignment with other related species. Moreover, tRNAs and their structures were identified by combining the results of ARWEN (Laslett and Canbäck 2008) and MITOS (Al Arab et al. 2017; Donath et al. 2019). The tRNAs, which were not detected, were obtained by comparing the sequence to *G. pseudorasborae* (Zeng et al. 2025). OGDRAW (version 1.3.1) was applied to generate a graphical diagram of the complete mitogenome (Greiner et al. 2019). The base composition, codon usage, and relative synonymous codon usage (RSCU) values were calculated with MEGA v12.0.2 (Kumar et al. 2024). The formula of AT‐skew and GC‐skew was used to analyze the base composition (Perna and Kocher 1995). Sliding window analysis was carried out in DnaSP v5 (Librado and Rozas 2009): a sliding window of 200 bp and a step size of 20 bp were applied to estimate the nucleotide divergence Pi between the mitogenomes of *G. nigeri* and other Gyrodactylidea species. Evolutionary rate analyses among the 12 PCGs of the mitogenomes of *G. nigeri* and other Gyrodactylidea species were conducted using KaKs_Calculator v. 2.0 with the MA method (Zhang et al. 2006). Tandem repeats in non‐coding regions were identified with Tandem Repeats Finder (Benson 1999), and their secondary structures were predicted by UNAFold software (Markham and Zuker 2008). The genomic synteny of the 14 mitogenomes (Table S1) was analyzed with Mauve v2.4.0 (Darling et al. 2004). Finally, the complete mitogenome sequence was deposited in the GenBank database with accession number PX562927.

### 2.5 Co‐Phylogenetic Analyses

Phylogenetic trees were performed using Bayesian inference (BI) and maximum likelihood (ML) methods based on PCGs nucleotide and amino acid sequences of the complete mitogenome of *G. nigeri* and twelve other Gyrodactylidea species and eight fish hosts of Cyprinidae, Nemacheilidae, and Cobitidae within Cypriniformes, Salmonidae within Salmoniformes, Cichlidae within Cichliformes, and Loricariidae within Siluriformes (Zardoya et al. 1995; Hurst et al. 1999; Huyse et al. 2007, 2008; Ye et al. 2014, 2017; Zou et al. 2016; Zhang et al. 2016; Bachmann et al. 2016; Moreira et al. 2017; Vanhove et al. 2018; Zhang, Li, et al. 2020; Janulewicz et al. 2024; Zeng et al. 2025) (Table 1). The oviparous flatworm *A. forficulatus* (KU679421) from the loricarid host *Kronichthys lacerta* Nichols, 1919 was used as an outgroup for gyrodactylids (Bachmann et al. 2016), as its host *K. lacerta* was not sequenced with mitogenome data. Therefore, the unique mitogenome from *Kronichthys heylandi* (Boulenger, 1900) (KT239014) was used as an outgroup for host fish (Moreira et al. 2017). The best‐fit models were determined using the Bayesian information criterion (BIC) in jModelTest v2.1.10 (Darriba et al. 2012), PhyloSuite v2 (Zhao et al. 2025), and ProtTest v3.4.2 (Darriba et al. 2011). The GTR + I + G and MtArt+G + F for gyrodactylids and GTR + I + G and MtMam+I + G for fish hosts were chosen as the best‐fitting models for BI and ML analyses. BI analysis was performed using MrBayes v3.2.7 (Ronquist et al. 2012), with one set of four chains running simultaneously for 10,000,000 generations. ML analysis was conducted using RAxML v8.2.10 (Stamatakis 2014), with bootstrap analysis performed with 1000 replicates.

**TABLE 1:** The information of protein‐coding genes (PCGs) nucleotide sequences of thirteen Gyrodactylidea species and eight fish hosts used for co‐phylogenetic analyses.

Species	GenBank number (parasite)	Host	GenBank number (host)	Host family	Host order	Locality	Parasitic site	References
*Gyrodactylus nigeri*	PX562927	*Yunnanilus niger*	MW553077	Nemacheilidae	Cypriniformes	Dalubei from Qujing City of Yunnan Province, China	Fins	This study
*Gyrodactylus pseudorasborae*	PP808686	*Pseudorasbora parva*	JF802126	Cyprinidae	Cypriniformes	Huixian wetland, Guilin City, China	Fins	Zeng et al. (2025)
*Gyrodactylus derjavinoides*	EU293891	*Oncorhynchus mykiss*	L29771	Salmonidae	Salmoniformes	Danish freshwater rainbow trout farm (Refsgaard, Jutland)	Laboratory culture	Zardoya et al. (1995), Huyse et al. (2008)
*Gyrodactylus gurleyi*	KU659806	*Carassius auratus*	KJ874430	Cyprinidae	Cypriniformes	Wuhan, China	Fins and gills	Zou et al. (2016)
*Gyrodactylus parvae*	KP780992	*Pseudorasbora parva*	JF802126	Cyprinidae	Cypriniformes	Cold‐water streams in Lantian County Lantian Counties, China	Skin	Ye et al. (2017)
*Gyrodactylus brachymystacis*	KT277549	*Oncorhynchus mykis*	L29771	Salmonidae	Salmoniformes	Fish farm located in Niubeiliang Nature Reserve Lantian Counties, China	Skin	Zardoya et al. (1995), Ye et al. (2017)
*Gyrodactylus kobayashii*	KU057942	*Carassius auratus*	KJ874430	Cyprinidae	Cypriniformes	Wuhan, China	Fins and gills	Zhang et al. (2016)
*Gyrodactylus salaris*	DQ988931	*Salmo salar*	U12143	Salmonidae	Salmoniformes	River Signal‐dalselva, North Norway	Undescribed	Hurst et al. (1999), Huyse et al. (2007)
*Gyrodactylus* sp. FY‐2015	KP780991	*Homatula variegata*	JX144893	Nemacheilidae	Cypriniformes	Qinling Mountain region of central China	Skin and fins	Unpublished
*Gyrodactylus* sp. FZ‐2021	MW464989	*Misgurnus anguillicaudatus*	MN116750	Cobitidae	Cypriniformes	Lancang River in Jinghong city, China	Gills	Zhang, Li, et al. (2020), Zhang, Zhang, et al. (2020)
*Cichlidarus nyanzae*	MG970256	*Oreochromis niloticus*	GU477625	Cichlidae	Cichliformes	Democratic Republic of the Congo: INERA station Kipopo	Gills	Vanhove et al. (2018), Janulewicz et al. (2024)
*Paragyrodactylus variegatus*	KM067269	*Homatula variegata*	JX144893	Nemacheilidae	Cypriniformes	Qinling Mountain region of central China	Skin and fins	Ye et al. (2014)
*Aglaiogyrodactylus forficulatus*	KU679421	*Kronichthys lacerta* (without mitogenome sequence)	KT239014 ( *Kronichthys heylandi* )	Loricariidae	Siluriformes	Brazil: Parana, Rio Morato, Microbacia Garaquecaba basin	External surfaces	Moreira et al. (2017), Bachmann et al. (2016)

Divergence time estimations of gyrodactylids and fish hosts based on molecular dating were performed in BEAST v1.10.4 (Suchard et al. 2018) based on PCGs nucleotide sequences. The divergence times were estimated using the GTR + I + G as a site model, an uncorrelated lognormal relaxed molecular clock model with a birth‐death speciation tree prior for host (Ritchie et al. 2017), and a strict molecular clock model with a Yule speciation tree prior for gyrodactylids. The mutation rate of 1% and 10% per million years ago (Mya) was adopted based on the mitochondrial gene in cyprinid fish (Durand et al. 2002; Chen et al. 2022) and a moderate rate in Monogenea (Li et al. 2011; Chen et al. 2020). Bayesian Markov Chain Monte Carlo (MCMC) analyses were performed for 10 million generations while sampling every 5000th tree, and the first 10% of the trees sampled were treated as burn‐in. Subsequently, the estimates and convergence of effective sample size (ESS) for all parameters larger than 200 were checked with Tracer v1.7.1 (Rambaut et al. 2018), and all resulting trees were combined with LogCombiner v1.10.4 (Suchard et al. 2018). Finally, a maximum credibility tree was produced using TreeAnnotator v1.10.4 (Suchard et al. 2018). Tanglegrams of gyrodactylids and fish hosts were visualized and annotated in iTOL v7.1 (Letunic and Bork 2024) to explore the co‐phylogenetic framework.

## 3 Results

### 3.1 Features of the Mitogenome and Gene Arrangement

**FIGURE 2:**
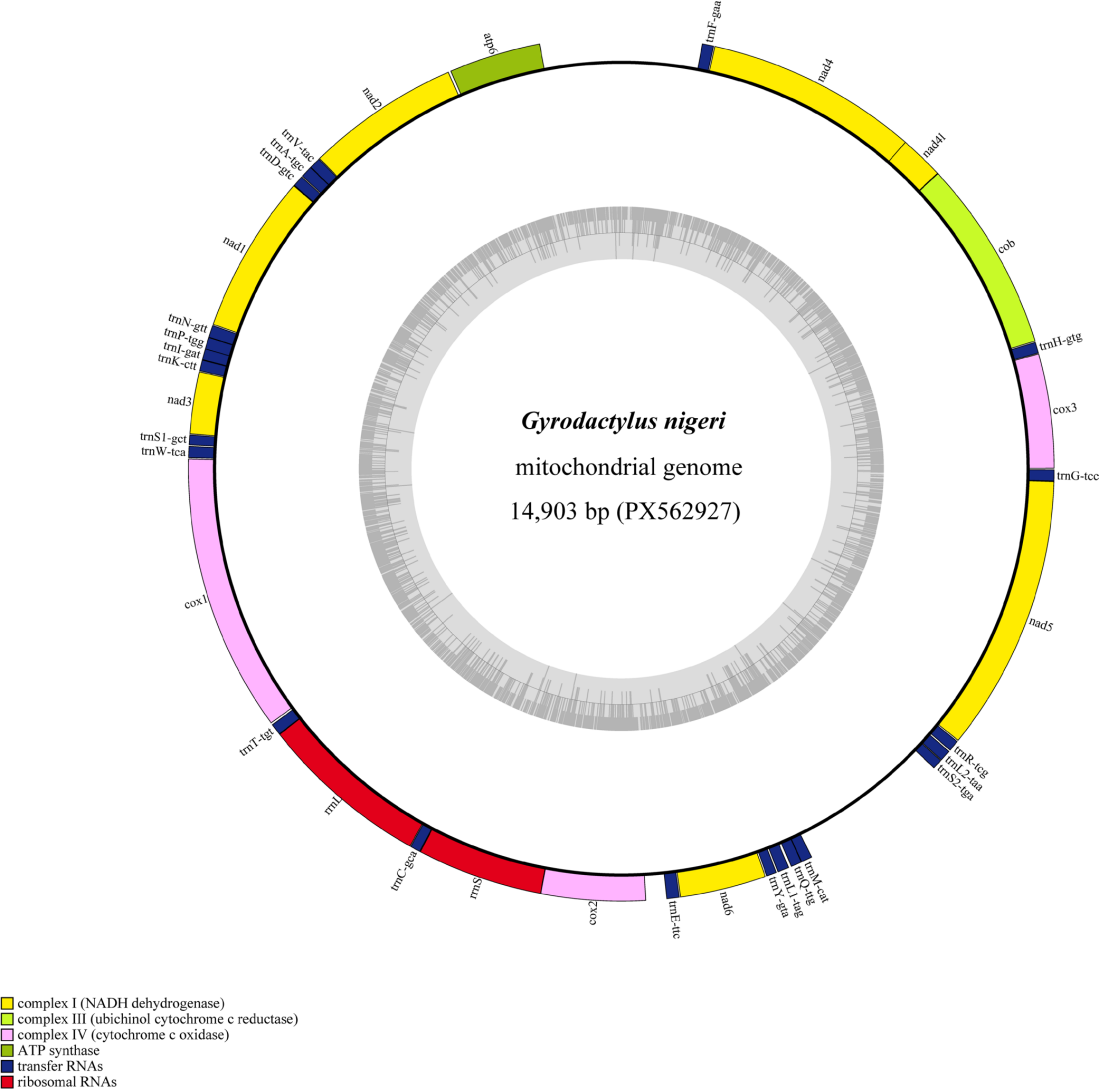
Graphical map of the mitogenome of *Gyrodactylus nigeri*. The yellow, pink, and green segments represent protein‐coding genes (PCGs); the blue and red segments represent tRNA genes (tRNAs) and rRNA genes (RNAs), respectively. Genes for tRNAs are abbreviated using three letters.

The circular mitogenome of *G. nigeri* (GenBank accession number PX562927) was 14,903 bp in length (Figure 2) and contained 12 protein‐coding genes (PCGs, lacking *Atp8*), 22 tRNA genes (tRNAs), two rRNA genes (rRNAs), and two major non‐coding regions (NCR: NC1 and NC2) (Table 2 and Figure 2). All the genes were transcribed from the same strand. The base composition of the whole mitogenome was 42.2% T, 9.2% C, 34.4% A, and 14.1% G (Table 3). The overall A + T content was up to 76.6%, which was approximate to *P. variegatus* from the Nemacheilidae hosts (Table 3 and Table S1). The AT‐skew values of *G. nigeri* were negative except for the NC2 region, while the GC‐skew values of *G. nigeri* were positive (Table 3). *G. nigeri* exhibited an obvious base T and G bias. The Gyrodactylidea species exhibit an obvious base T and G bias in whole mitogenomes and PCGs (Tables S1 and S2).

**TABLE 2:** Organization of the mitochondrial genome of *Gyrodactylus nigeri*.

Gene/region	Position		Size	Intergenic nucleotides	Codon		Anti‐codon
	From	End			Start	Stop	
*Cox3*	1	639	639		ATG	TAA	
*tRNA* ^*His*^	641	705	65	1			GTG
*Cytb*	709	1782	1074	3	ATG	TAA	
*Nad4l*	1782	2030	249	−1	ATG	TAA	
*Nad4*	2003	3211	1209	−28	ATG	TAA	
*tRNA* ^*Phe*^	3215	3280	66	3			GAA
*NC1*	3281	4174	894				
*Atp6*	4175	4687	513		ATG	TAA	
*Nad2*	4699	5556	858	11	ATG	TAA	
*tRNA* ^*Val*^	5558	5624	67	1			TAC
*tRNA* ^*Ala*^	5623	5691	69	−1			TGC
*tRNA* ^*Asp*^	5694	5758	65	2			GTC
*Nad1*	5759	6646	888		ATG	TAA	
*tRNA* ^*Asn*^	6650	6720	71	3			GTT
*tRNA* ^*Pro*^	6719	6788	70	−2			TGG
*tRNA* ^*Ile*^	6784	6848	65				GAT
*tRNA* ^*Lys*^	6848	6911	64	−1			CTT
*Nad3*	6914	7261	348	4	ATG	TAA	
*tRNA* ^*Ser(AGN)*^ (S1)	7264	7322	59				GCT
*tRNA* ^*Trp*^	7327	7393	67	4			TCA
*Cox1*	7397	8944	1548	4	ATG	TAA	
*tRNA* ^*Thr*^	8953	9019	67	8			TGT
*rrnL*	9019	9969	951	2			
*tRNA* ^*Cys*^	9971	10,030	60	1			GCA
*rrnS*	10,031	10,733	703	2			
*Cox2*	10,732	11,313	582		ATG	TAA	
*tRNA* ^*Glu*^	11,425	11,497	73	111			TTC
*Nad6*	11,501	11,983	483	2	ATG	TAA	
*tRNA* ^*Tyr*^	11,986	12,047	62	5			GTA
*tRNA* ^*Leu(CUN)*^ (L1)	12,054	12,120	67	6			TAG
*tRNA* ^*Gln*^	12,133	12,196	64	12			TTG
*tRNA* ^*Met*^	12,195	12,258	64	−2			CAT
*NC2*	12,259	13,088	830				
*tRNA* ^*Ser(UCN)*^ (S2)	13,089	13,147	59				TGA
*tRNA* ^*Leu(UUR)*^ (L2)	13,149	13,214	66	1			TAA
*tRNA* ^*Arg*^	13,221	13,285	65	6			TCG
*Nad5*	13,290	14,834	1545		ATG	TAA	
*tRNA* ^*Gly*^	14,834	14,899	66	−1			TCC
	14,900	14,903		4			

**TABLE 3:** Nucleotide composition and skewness comparison of different elements of the mitochondrial genome of *Gyrodactylus nigeri*.

Regions	Size (bp)	T (U) (%)	C (%)	A (%)	G (%)	AT (%)	GC (%)	AT skew	GC skew
Complete genome	14,903	42.2	9.2	34.4	14.1	76.6	23.4	−0.102	0.209
PCGs	9936	44.1	8.9	32.8	14.2	76.9	23.1	−0.147	0.230
1st codon position	3312	39.3	8.1	34.7	17.9	74.0	26.0	−0.062	0.377
2st codon position	3312	49.3	11.7	20.6	18.4	69.9	30.1	−0.411	0.223
3st codon position	3312	43.6	7.0	43.1	6.4	86.7	13.3	−0.006	−0.045
*Atp6*	513	46.0	9.2	30.6	14.2	76.6	23.4	−0.201	0.217
*Cox1*	1548	42.6	11.1	29.3	17.0	71.9	28.1	−0.186	0.209
*Cox2*	582	40.4	11.5	31.4	16.7	71.8	28.2	−0.124	0.183
*Cox3*	639	43.2	9.4	32.9	14.6	76.1	23.9	−0.136	0.216
*Cytb*	1074	41.4	9.8	31.5	17.3	72.9	27.1	−0.137	0.278
*Nad1*	888	46.7	8.7	30.7	13.9	77.5	22.5	−0.206	0.230
*Nad2*	858	44.6	6.9	37.0	11.5	81.6	18.4	−0.094	0.253
*Nad3*	348	50.0	5.5	30.8	13.8	80.8	19.3	−0.238	0.433
*Nad4*	1209	45.2	7.4	33.8	13.7	78.9	21.1	−0.145	0.294
*Nad4l*	249	48.6	7.2	32.9	11.2	81.5	18.5	−0.192	0.217
*Nad5*	1545	43.8	8.4	36.3	11.5	80.1	19.9	−0.095	0.156
*Nad6*	483	43.7	8.7	35.2	12.4	78.9	21.1	−0.108	0.176
*rrnL*	951	40.5	9.8	36.8	12.9	77.3	22.7	−0.048	0.139
*rrnS*	704	36.6	10.4	39.1	13.9	75.7	24.3	0.032	0.146
rRNAs	1654	38.8	10.0	37.8	13.4	76.6	23.4	−0.013	0.142
tRNAs	1438	40.0	9.3	37.3	13.4	77.3	22.7	−0.035	0.180
NC1	894	37.4	10.2	36.9	15.5	74.3	25.7	−0.006	0.208
NC2	796	36.7	11.2	36.7	15.5	73.4	26.6	0.000	0.160

**FIGURE 3:**
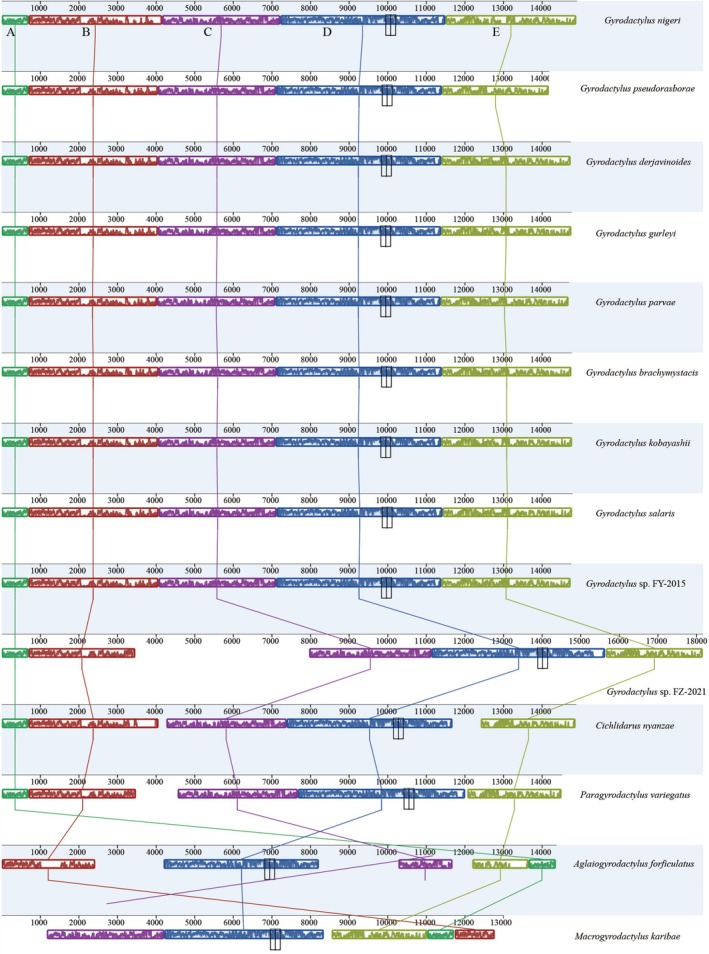
Gene orders and gene synteny analysis are shown among 14 mitogenomes of Gyrodactylidea species. The rearrangements of tRNAs and PCGs occurred in five gyrodactylids, including *Gyrodactylus* sp. FZ‐2021, *Cichlidarus nyanzae*, *Paragyrodactylus variegatus*, *Macrogyrodactylus karibae*, and *Aglaiogyrodactylus forficulatus*. Gene synteny analysis revealed five homologous regions (A–E).

The gene order of *G. nigeri* was identical to that of *Gyrodactylus* species except for *Gyrodactylus* sp. FZ‐2021 (Table 2 and Figure 3), and the rearrangements of tRNAs and PCGs were consistent with five other gyrodactylids, including *Gyrodactylus* sp. FZ‐2021, *C. nyanzae* , *P. variegatus* , *M. karibae*, and *A. forficulatus* in a previous study (Zeng et al. 2025) (Figure 3). Gene synteny analysis revealed five homologous regions (A‐E) in 14 mitogenomes of Gyrodactylidea (Figure 3). The relative positions and sizes of PCGs in *G. nigeri* and the other 12 species were highly conserved, while *M. karibae* and *A. forficulatus* were rearrangements from two to three homologous regions (A, B, and C) corresponding to two to three PCG genes, the mitogenome sequence of *Gyrodactylus* sp. FZ‐2021 was longer than that of other mitogenomes (Figure 3).

The length of the 22 tRNA genes varied from 59 bp (*tRNA* ^*Ser(AGN*)^ (S1) and *tRNA* ^*Ser(UCN)*^ (S2)) to 73 bp (*tRNA* ^*Glu*^) (Table 2). All tRNAs could fold into the conventional secondary structure, except for three unorthodox tRNAs, *tRNA* ^*Ser(AGN)*^, *tRNA* ^*Ser(UCN)*^, and *tRNA* ^*Cys*^ lacked the dihydrouridine (DHU) arms. The sizes of *rrnL* and *rrnS* were 951 bp with 77.3% and 703 bp with 75.7% A + T content, respectively (Table 3), they were separated by *tRNA* ^*Cys*^ (Table 2). Two *Gyrodactylus* species (*G. derjavinoides* and *G. salaris* ) and seven Gyrodactylidae species (*G. pseudorasborae*, *G. derjavinoides*, *G. parvae*, *G. brachymystacis*, *G. salaris* , *Gyrodactylus* sp. FY‐2015, and *P. variegatus* ) showed positive AT‐skew values for the rRNAs and NCR, respectively (Tables S3 and S4).

**FIGURE 4:**
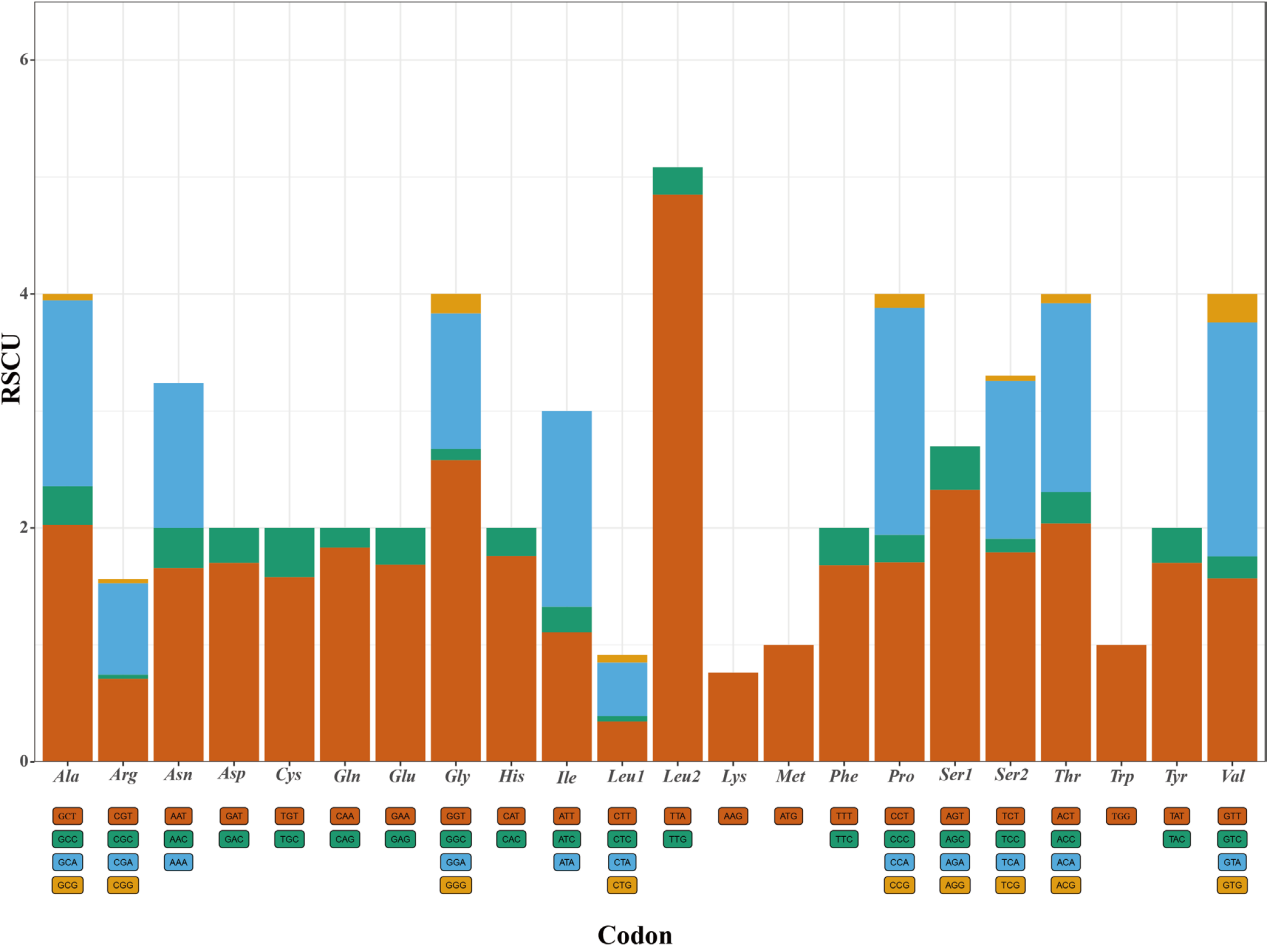
Codon and relative synonymous codon usage (RSCU) values of the protein‐coding genes (PCGs) in the mitochondrial genome of *Gyrodactylus nigeri*.

All PCGs used the canonical ATG as the start codon and the canonical stop codons TAA (Table 2). The length of 12 PCGs was 9936 bp, with 76.9% A + T content (Table 3). Furthermore, the incomplete stop codon TA was observed in gyrodactylid *G. brachymystacis* (Table S5). The codon usage and RSCU values are summarized (Figure 4). The most RSCU values of amino acids in the PCGs of *G. nigeri* are Serine (Ser), Leucine (Leu), and Threonine (Thr), the lowest in Methionine (Met) and Lysine (Lys) (Figure 4). In addition, there were eight cases of overlapping regions within the mitogenome. The overlap between *Nad4l* and *Nad4* was common in metazoan mtDNAs. There were 21 cases of short intergenic areas ranging from 1 bp to 111 bp (Table 2).

### 3.2 Non‐Coding Regions

**FIGURE 5:**
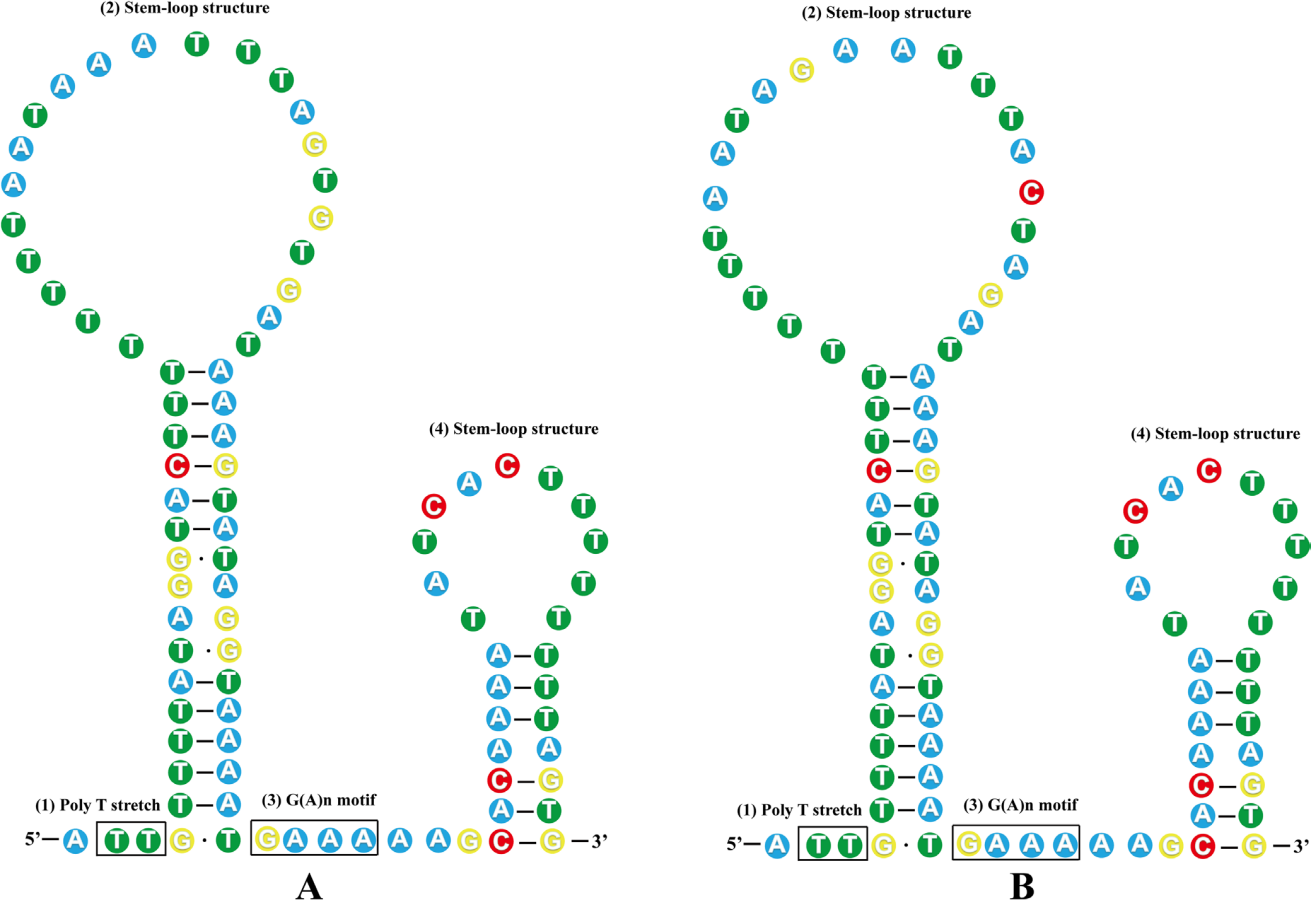
Stem‐loop structural elements of two repeat patterns for the mitochondrial non‐coding regions (NCR) of *Gyrodactylus nigeri*.

The two long non‐coding regions, NC1 (between *tRNA* ^*Phe*^ and *Atp6*) and NC2 (between *tRNA* ^*Met*^ and *tRNA* ^*Ser(UCN)*^) were 894 bp and 830 bp in size (Table 2 and Figure 1), with 74.3% and 73.4% A + T content, respectively (Table 3). There were two repetitive regions with a consensus pattern (88 bp) in NC1 and NC2 regions with 7 bp differences, and they could form a stem‐loop structure with a poly‐T stretch, two stem‐loop structures with obvious differences in the first loop, and a G(A)n motif (Figure 5).

### 3.3 Nucleotide Diversity and Evolutionary Rate

**FIGURE 6:**
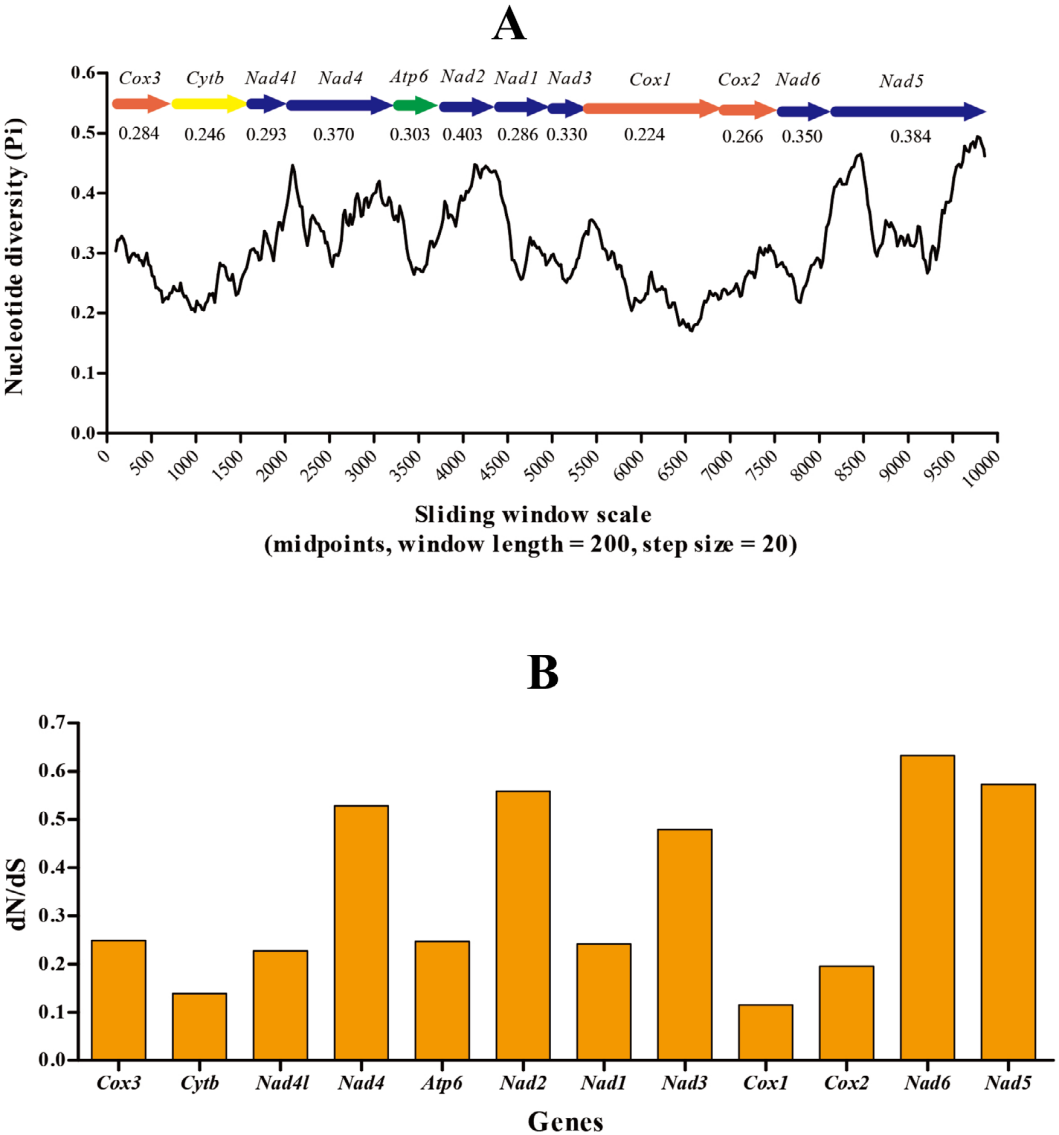
Sliding window and evolutionary rate analyses of the PCGs of mitogenomes among twelve Gyrodactylidea species. (A) Sliding window analysis was conducted on concatenated alignments of 13 PCGs. The black line represents the value of nucleotide diversity (window size = 200 bp, step size = 20 bp, with the value inserted at its midpoint). Gene names, boundaries, and average nucleotide diversity values are indicated above the graph. (B) Ratios of non‐synonymous (dN) to synonymous (dS) substitution rates calculated for protein‐coding genes (PCGs).

The plot of sequence variation ratio exhibited highly variable nucleotide diversity, with Pi values for the 200 bp windows ranging from 0.182 to 0.494 (Figure 6A). Genes with comparatively high sequence variability were *Nad2* (0.403), *Nad5* (0.384), *Nad4* (0.370), and *Nad6* (0.350), whereas *Cox1* (0.224) and *Cytb* (0.246) had comparatively low sequence variability. The non‐synonymous/synonymous (dN/dS) ratio analysis showed that all PCGs are under purifying selection and evolving under comparatively relaxed mutational constraints (Figure 6B).

### 3.4 Co‐Phylogenetic Analyses

**FIGURE 7:**
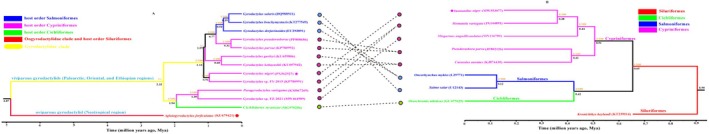
The tanglegrams of gyrodactylids and fish hosts inferred using the protein‐coding genes (PCGs) nucleotide dataset. The figure contains a parasite tree (A) with its family‐level and the host order‐level annotations, a host tree (B) with the order‐level annotation, and a set of host–parasite associations (the host range of each gyrodactylid). The scale bar represents the estimated divergence time. Node numbers above represent the Bayesian posterior probabilities and bootstrap values (BI/ML), respectively (those above 50% are shown). Estimated divergent dates in million years ago (Mya) are given in numbers down nodes. The study gyrodactylid with its host and outgroup species are highlighted by a magenta pentagram and a red circle, respectively.

Co‐phylogenetic analyses based on PCGs datasets produced robust and congruent phylogenetic trees of gyrodactylids (Figure 7A) and fish hosts (Figure 7B) with identical branch topologies and only minor differences in Bayesian posterior probabilities and bootstrap values for two nodes in gyrodactylids, and the overall coevolutionary fit between the parasites and hosts was consistently significant (Figure 7). The results supported the sister relationship between *G. nigeri* and *Gyrodactylus* sp. FY‐2015 with maximum support (both BI = 1, both ML = 100) from two hosts within the Nemacheilidae, and the Gyrodactylidae formed an independent clade; the genus *Gyrodactylus*, originally hosted by cyprinids, didn't form an independent clade (Table 1, Figure 7). Furthermore, tanglegrams showed that gyrodactylids grouped in different clades were associated with fish host orders Cypriniformes, Salmoniformes, and Cichliformes; the early‐diverging host lineages tend to be parasitized by early‐diverging parasite lineages (Figure 7).

## 4 Discussion

### 4.1 Mitogenome Characteristics and Evolution

Compared with the Gyrodactylidea mitogenomes, *G. nigeri* showed a higher A + T content, the same gene order as most *Gyrodactylus* species, *tRNA* ^*Ser*(*AGN*)^, *tRNA* ^*Ser*(*UCN*)^, and *tRNA* ^*Cys*^ without the DHU arms, and two tandem repeat sequences within the NCR formed two stem‐loop structures. Co‐phylogenetic analyses consistently supported that *G. nigeri* is a member of the genus *Gyrodactylus* and Gyrodactylidae forms an independent monophyletic clade within Gyrodactylidea. Phylogenetic divergence patterns of *Gyrodactylus* correspond to those of their fish hosts. Due to the limited resolution of our datasets, it is necessary to sequence more molecular data, such as mitogenomes, transcriptomes, and multiple nuclear genes, to resolve the deep phylogeny of Gyrodactylidea and co‐phylogenetic patterns between gyrodactylids and fish hosts.

The complete mitogenome of *G. nigeri* also contains 36 common flatworm mitochondrial genes (Wey‐Fabrizius et al. 2013). It exhibits a higher A + T content within the order Gyrodactylidea, ranging from 62.5% ( *G. salaris* ) (Huyse et al. 2007) to 80.1% ( *C. nyanzae* ) (Vanhove et al. 2018; Janulewicz et al. 2024), while the mitogenome of *Paratetraonchoides inermis* Bychowsky, Gussev & Nagibina, 1965 was the highest A + T content (82.6%) among the monogenean (Zhang et al. 2017). In the monogenean, TTG was also used as a start codon for *Tetraonchus monenteron* (Wagener, 1857), Diesing, 1858 (Zhang et al. 2020), *C. nyanzae*, and *M. karibae* (Vanhove et al. 2018; Janulewicz et al. 2024), and *P. variegatus* (Ye et al. 2014) in *Nad41* and *Cox2*, respectively (Table S5), while GTG was also used as a start codon for *P. inermis* (Zhang et al. 2020), *Gyrodactylus* sp. FZ‐2021 (MW464989), *C. nyanzae* and *M. karibae* (Vanhove et al. 2018; Janulewicz et al. 2024), and *P. variegatus* (Ye et al. 2014) in *Cox1* and *Nad1*, respectively (Table S5). It was also proposed as an alternative start codon for flatworm mitogenomes (Ross et al. 2016). The second position of the PCGs exhibited a strong preference for base T of *G. nigeri* and was consistent with the Gyrodactylidea species without *Gyrodactylus* sp. FZ‐2021 (MW464989) and *P. variegatus* (Ye et al. 2014). This is also the argument that codons for hydrophobic amino acid residues, which are functionally preferred for conformational stability of mitochondrial proteins, mostly have T in the second codon position (Naylor et al. 1995). The third position of the PCGs exhibited a strong preference for T of *Gyrodactylus* sp. FZ‐2021 (MW464989) and *P. variegatus* (Ye et al. 2014) and affects translation speed and the folding of the nascent protein (Stadler and Fire 2011; Schaffrath and Leidel 2017).

The present study confirms the same trends of the RSCU and dN/dS values with order Gyrodactylidea species (Huyse et al. 2007, 2008; Ye et al. 2014, 2017; Zou et al. 2016; Zhang et al. 2016, 2017; Zhang, Zhang, et al. 2020; Bachmann et al. 2016; Vanhove et al. 2018; Zeng et al. 2025). All tRNAs could fold into the conventional secondary structure, except for three unorthodox tRNAs, *tRNA* ^*Ser*(*AGN*)^, *tRNA* ^*Ser*(*UCN*)^, and *tRNA* ^*Cys*^, which lacked the DHU arms within the Order Gyrodactylidea (Huyse et al. 2007, 2008; Ye et al. 2014, 2017; Zou et al. 2016; Zhang et al. 2016; Bachmann et al. 2016; Vanhove et al. 2018; Zeng et al. 2025), and *tRNA* ^*Pro*^ lacked the TΨC arm in *G. salaris* (Huyse et al. 2007). In addition, two unorthodox tRNAs, *tRNA* ^*Ser*(*AGN*)^ and *tRNA* ^*Cys*^, also lacked DHU arms in *P. inermis* (Tetraonchoididae) (Zhang et al. 2017).

Similar repetitive regions and stem‐loop structures formed by their tandem repeats were also found in NCR located in different intergenic regions within Gyrodactylidea, including *G. gurleyi* (Zou et al. 2016), *Gyrodactylus* sp. FZ‐2021 (unpublished), *G. pseudorasborae* (Zeng et al. 2025), *C. nyanzae* (Vanhove et al. 2018; Janulewicz et al. 2024), *P. variegatus* (Ye et al. 2014), and *A. forficulatus* (Bachmann et al. 2016). The presence of tandem repeats forming a stable stem‐loop structure is usually associated with the replication origin in mitochondria (Le et al. 2002; Fumagalli et al. 1996; Ye et al. 2014; Zou et al. 2016; Bachmann et al. 2016; Vanhove et al. 2018; Janulewicz et al. 2024; Zeng et al. 2025), which suggests that NCR may be involved in the initiation of replication of the mitogenome of *G. nigeri*. The overlap between *Nad4L* and *Nad4* was common in metazoan mtDNAs (von Nickisch‐Rosenegk et al. 2001), except *Benedenia hoshinai* Ogawa, 1984 and *B. seriolae* (Yamaguti 1934) Meserve, 1938 (Perkins et al. 2010). The *G. nigeri* was the third sequenced mitogenome compared with *Gyrodactylus* sp. FY‐2015 (KP780991) and *P. variegatus* from different hosts within Nemacheilidae in China (Ye et al. 2014). While the main differences between *G. nigeri* and *Gyrodactylus* sp. FY‐2015 (KP780991) was in the length and base composition of the NCR of the mitogenome.

The sliding window and evolutionary rate analyses also showed the *Cox1* gene is the slowest‐evolving and most conserved in population genetics studies of monogeneans (Hansen et al. 2003, 2006; Blasco‐Costa et al. 2012; Ye et al. 2014, 2017; Vanhove et al. 2018; Zhang, Li, et al. 2020; Janulewicz et al. 2024; Zeng et al. 2025). On the contrary, the faster‐evolving *Nad2* and *Nad5* genes may be better molecular markers for species and population‐level genetics studies of the Gyrodactylidea and capable of providing higher resolution for phylogenetically closely related taxa and populations (Goldstein et al. 1995; Ye et al. 2014, 2017; Zhang et al. 2017; Zhang, Zhang, et al. 2020).

### 4.2 Gene Order and Arrangement

The result showed that the gene order and gene synteny of *G. nigeri* were the same as most *Gyrodactylus* species (Zeng et al. 2025), meanwhile, extensive rearrangements of tRNAs also exhibited in other Gyrodactylidea and Tetraonchidea species (Ye et al. 2014; Bachmann et al. 2016; Vanhove et al. 2018; Zhang et al. 2017; Zhang, Li, et al. 2020; Janulewicz et al. 2024; Zeng et al. 2025). Minor rearrangements of PCGs exhibited in viviparous *M. karibae* in Africa (Vanhove et al. 2018; Truter et al. 2021) and oviparous *A. forficulatus* in South America (Kritsky et al. 2007; Bachmann et al. 2016). The unique tRNA gene orders (Q‐M‐F) and (Y‐L1‐S2‐L2) were also found in Gyrodactylidea and Tetraonchidea species (Ye et al. 2014; Bachmann et al. 2016; Vanhove et al. 2018; Zhang et al. 2017; Zhang, Zhang, et al. 2020; Janulewicz et al. 2024; Zeng et al. 2025). The most extensive rearrangements of tRNAs and PCGs occurred in an oviparous *A. forficulatus* (Bachmann et al. 2016; Zeng et al. 2025). Although gene order can be used to infer phylogenetic relationships (Boore 2006; Sultana et al. 2013), this discontinuity in rearrangements and the limited information content of gene order in monogeneans might produce misleading evolutionary signals and be used for resolving relationships at shallow taxonomic scales.

### 4.3 Co‐Phylogenetic Framework

The co‐phylogenetic results support that *G. nigeri* forms a well‐supported sister relationship with *Gyrodactylus* sp. FY‐2015 from different hosts within Nemacheilidae in China, and belongs to the genus *Gyrodactylus* (Figure 7) (Zhou and Chen 2024), and the coevolutionary fit between gyrodactylids (Figure 7A) and their hosts (Figure 7B) was highly significant (Lei et al. 2024). The phylogenetic divergence patterns of *Gyrodactylus* originally hosted within Cypriniformes correspond to those of their fish hosts, and later, divergence to Salmoniformes by host switch based on divergence time (Figure 7). Our analyses also support host switch events, and phylogeny affected the coevolution of gyrodactylids and their fish hosts (Lei et al. 2024). Gyrodactylidae formed an independent monophyletic clade within Gyrodactylidea and included viviparous gyrodactylids from Palearctic, Oriental, and Ethiopian regions, which is consistent with previous studies based on several mitogenomes, mitochondrial and nuclear genes (Bachmann et al. 2016; Vanhove et al. 2018; Zhang, Li, et al. 2020; Boeger et al. 2021; Zeng et al. 2025), and the genus *Gyrodactylus* is not monophyletic (Zhang et al. 2017; Janulewicz et al. 2024; Zeng et al. 2025). As *Gyrodactylus* sp. FZ‐2021 from host *Misgurnus anguillicaudatus* (Cantor, 1842) lacked morphological characteristics description and rearrangements of tRNAs, meanwhile, many *Gyrodactylus* species from *M. anguillicaudatus* have been revised new genus *Macracanthus* Janulewicz, Pietkiewicz & Zietara, 2024, such as *Macracanthus granoei* (You et al. 2010) Janulewicz, Pietkiewicz & Ziętara, 2024, *Macracanthus jennyae* (Paetow et al. 2009) Janulewicz, Pietkiewicz & Ziętara, 2024, and *Macracanthus macracanthus* (Hukuda 1940) Janulewicz, Pietkiewicz & Ziętara, 2024, therefore, *Gyrodactylus* sp. FZ‐2021 might belong to the genus *Macracanthus*. Furthermore, gyrodactylids grouped in different clusters are associated with fish host families Cyprinidae, Nemacheilidae, Cobitidae, Salmonidae, and Cichlidae, and orders Cypriniformes, Salmoniformes, and Cichliformes (Zeng et al. 2025). The genus *Gyrodactylus* in this study showed high host specificity (mainly infected a single host) with limited species (Zietara and Lumme 2002; Whittington et al. 2000; Harris et al. 2004; Lei et al. 2024). While the partly cultured Salmonidae species and goldfish *Carassius auratus* (Linnaeus, 1758) may have existed more than two *Gyrodactylus* species, especially, the *G. salaris* occurred in 12 kinds of fish hosts (Harris et al. 2004), the main reason may be affected by host switch between coexisting hosts in the same geographic distribution (Harris et al. 2004; Lei et al. 2024). It would be better to sequence more mitogenome data to infer Gyrodactylidae phylogeny and test coevolution between gyrodactylids and fish hosts.

## Author Contributions

**Yuhan Yang:** data curation (equal), formal analysis (equal), project administration (equal), validation (equal), visualization (equal). **Yang Liu:** data curation (equal), formal analysis (equal), project administration (equal), supervision (equal), validation (equal). **Wenhan Yue:** data curation (equal), project administration (equal), validation (equal), visualization (equal). **Yuxuan Chen:** data curation (equal), project administration (equal), validation (equal), visualization (equal). **Man Kang:** conceptualization (equal), funding acquisition (equal), resources (equal). **Yulin He:** methodology (equal). **Tao Chen:** conceptualization (equal), funding acquisition (equal), resources (equal), supervision (equal), visualization (equal), writing – original draft (equal), writing – review and editing (equal).

## Funding

This work was funded by the Guangxi Natural Science Foundation (grant number 2025GXNSFAA069864), School Enterprise Cooperation Program (grant number 20401023004), and the College Students’ Innovative Entrepreneurial Training Plan Program (grant number 202310601023).

## Conflicts of Interest

The authors declare no conflicts of interest.

## Supporting information


**Table S1:** Nucleotide composition and skewness comparison of the complete mitochondrial genomes of 14 species in Gyrodactylidea.

**Table S2:** Nucleotide composition and skewness comparison of PCGs of the complete mitochondrial genomes of 13 species in Gyrodactylidea.

**Table S3:** Nucleotide composition and skewness comparison of rRNAs of the complete mitochondrial genomes of 14 species in Gyrodactylidea.

**Table S4:** Nucleotide composition and skewness comparison of NCR of the complete mitochondrial genomes of 13 species in Gyrodactylidea.

**Table S5:** Start and stop codons of PCGs of the complete mitochondrial genomes of 13 species in Gyrodactylidea.

## Data Availability

All the required data are uploaded as Supporting Information. The complete mitochondrial genome of *Gyrodactylus nigeri* was deposited in the GenBank of NCBI (https://www.ncbi.nlm.nih.gov) and obtained the accession number (GenBank accession no. PX562927). The data has been uploaded into Dryad under the following https://doi.org/10.5061/dryad.0k6djhbfs.
